# Mass spectrometry in clinical protein laboratories

**DOI:** 10.1515/almed-2024-0075

**Published:** 2024-06-13

**Authors:** Carmen Mugueta, Alvaro González, Sara Deza, Cristina Agulló Roca, Teresa Contreras, Noemí Puig, Nerea Varo

**Affiliations:** Service of Biochemistry, Clínica Universidad de Navarra, Pamplona, Spain; 37479 Hospital Universitario de Salamanca, Salamanca, Castilla y León, Spain

**Keywords:** mass spectrometry, multiple myeloma, monoclonal gammopathies

Joseph John Thomson was an engineer and mathematician who was awarded the Nobel Prize in Physics for his discovery of the electron in 1906, the same year that Santiago Ramón y Cajal received the Nobel Prize in Medicine. As early as in 1899, Thompson already described an instrument that resembled a mass spectrometer. Indeed, in the following decade, the first modern mass spectrometers were developed by his disciples Aston and also Dempster, from the University of Chicago. Since then, we have witnessed extraordinary technological advances, starting with the introduction of quadrupole time-of flight instruments. The electrospray solved the problem of large protein ionization and expanded the range of analysis, which was initially limited to small compounds. In his lecture for the Nobel Prize in Chemistry held in 2002, Fenn stated that it was *“to give electrospray wings to molecular elephants”.* These and subsequent improvements, such as Matrix Assisted Laser Desorption/Ionization (MALDI) and ionic trapping, turned mass spectrometry (MS) into a powerful, versatile, precise and sensitive analytical tool whose use has spread to a variety of scientific fields, including the clinical laboratory. To date, routine use of MS in the clinical laboratory has been limited to drug, steroid hormone and other metabolite testing. However, MS has a wide range of potential applications due to its characteristics. Indeed, in the last years, its use has spread to the analysis of large molecules such as proteins, including monoclonal immunoglobulins, which are used as biomarkers for the diagnosis and monitoring of monoclonal gammopathies (MGs).

The paradigm of malignant MG is multiple myeloma (MM), which is the second most frequent hematologic cancer. MM develops as a result of an excessive proliferation of plasma cells, which generally produce and secrete a monoclonal immunoglobulin that is detectable in serum and/or urine and whose concentration reflects tumor burden. A variety of drugs and therapeutic strategies have been developed in the recent years (proteasome inhibitors, immunomodulators and high dose chemotherapy followed by autologous transplant, to name a few) that with immunotherapy (monoclonal, bispecific antibodies and CAR-T cells), substantially improve the prognosis of MM. Currently, MM patients often achieve optimal response – i.e. complete response (CR) as assessed by conventional methods, most frequently after receiving the first line of treatment. CR is attained when the monoclonal protein (MP) is undetectable in serum and urine by electrophoresis and immunofixation and levels of clonal plasma cells in the bone marrow (BM) are <5 %. Although CR rates have increased dramatically with the new treatments, unfortunately, patients often relapse. This is caused by the persistence of disease, which remains undetectable under conventional response assessment methods. Disease “beyond CR” is known as “minimal residual disease” (MRD). In accordance with the recommendations of the *International Myeloma Working Group* (IMWG), screening for MRD should be performed in patients who have achieved CR.

The presence of MRD in the BM can be detected through the identification of residual plasma cells in the bone marrow or the secreted monoclonal proteins into the circulation. The presence of disease outside the BM (extramedullary disease) should be investigated by PET/CT. In 2016, the IMWG released consensus criteria for “negative MRD” for these techniques in order to standardize and enable the comparison of clinical studies. There is cumulative evidence that negative MRD is one of the factors with the highest prognostic value in MM (both at diagnosis and at relapse). In addition, it has been demonstrated that negative MRD is associated with significantly longer free-of disease and overall survival rates.

Although the clinical value of screening the BM for MRD or through PET/CT scanning is undeniable, these techniques also have some limitations. The analysis of BM by NGF or NGS requires obtaining a sample of the BM by aspiration (or biopsy), a painful, expensive, invasive procedure that cannot be repeated with the frequency required for adequate monitoring. Additionally, analysis is performed in a single sample, which may not be representative of medullary cellularity and the actual tumor burden (due to hemodilution, the typical patchy MM infiltration or the presence of extramedullary disease); therefore, false negative results may be obtained. Moreover, PET/CT scanning cannot be repeated frequently to avoid patient’s exposure to excessive radiation. In addition, results may be difficult to interpret, and the study has not yet been completely standardized. Finally, false negative results may be obtained due to enzymatic defects in tumor plasma cells that hamper tracer uptake. For these reasons, further research is needed to investigate the use of alternative samples. An example is peripheral blood, which is easy to collect and can be tested with an optimal frequency using highly-sensitive techniques. As compared to conventional techniques, the use of these samples will provide results with a higher clinical value or that are comparable or complementary to BM analysis.

To overcome these limitations, MS emerges as a useful instrument for the detection of MP in serum. This technique has shown better analytical performance than the methods currently used for this purpose in the clinical laboratory [1, 2]. MS can reach a sensitivity up to 1,000 times higher than electrophoresis. It has a very high specificity, as the mass/charge ratio of the MP is unique and specific to each patient. MS has a variety of applications for the diagnosis and follow-up of patients with MM and other GMs. Firstly, MS differentiates the endogenous tumor MP of the patient from therapeutic monoclonal antibodies, which form part of the standard treatment for all cases of MM. These therapeutic monoclonal antibodies, being the majority of the IgG kappa type, may interfere with the MP of the patient and hamper or hinder appropriate response evaluation. Certainly, there are methods available for eliminating interferences from particular therapeutic antibodies. However, it is unfeasible that a specific method is available in the clinical laboratory for eliminating each potential interfering therapeutic monoclonal antibody. MS, whereby compounds are identified based on their specific mass/charge, makes it possible to unequivocally separate therapeutic monoclonal antibodies from patient’s endogenous MP. Therefore, MS emerges as a universal solution to this significant analytical challenge.

Another use of MS is based on its higher sensitivity in identifying the presence of a MP in serum, as compared to conventional methods [2, 3]. Recent studies demonstrate that the presence of MP can be detected by MS in approximately 20 % of patients with negative results in conventional studies (or in CR). The detection of MP by MS is associated with a lower progression-free survival. Indeed, the IMWG accepts the use of MS instead of immunofixation both, in routine practice and in clinical trials [4]. As compared to NGF in BM, MS has been proven to have a slightly lower sensitivity but a clinical value comparable to those of conventional methods; therefore, at some points of the treatment, MS could provide complementary information to the analysis of BM for MRD. Although further data is needed, as MS testing is performed in serum, it can be performed at a higher frequency; therefore, it can help determine the optimal timing of MRD screening, thereby reducing the number of BM aspirations and optimizing the clinical usefulness of results. MS makes it possible to identify the presence of PM and monitor its evolution after treatment in patients categorized as “non-secretors” by conventional techniques. Should this be confirmed, the implantation of MS would considerably facilitate patient monitoring (which otherwise has to be performed through BM aspiration and/or PET/CT). Consequently, these patients could be included in clinical trials, from which they are generally excluded, in the absence of measurable disease.

On another note, MS provides additional clinically-valuable information about MP in patients with MM and other GMs that cannot be obtained through standard methods. More specifically, MS detects the presence of light chain glycosylation as a post-translational modification, which is considered a risk factor for progression in patients with MGUS and points to certain diagnoses such as light chain amyloidosis or cold agglutinin disease.

MS-based detection of MP in patients with MM and other GMs is a promising analytical option, in the light of the evidence currently available supporting its clinical value throughout the course of the disease. However, its implementation is not exempt from challenges. The higher sensitivity of MS, as compared to conventional electrophoresis, may lead to new findings of unknown clinical significance resulting in an increase in healthcare activity and demand for monitoring tests. In addition, as it has a higher sensitivity, CR rates will decrease with the use of MS. Therefore, direct comparison of CR as determined by conventional methods and by MS should be performed with caution. Finally, it is necessary that the use of MS goes beyond highly-specialized and research laboratories for it to be introduced into routine practice in a robust way. For that purpose, further commercial efforts should be made to develop automated, optimized MS techniques with capacity to analyze a higher volume of samples and that software is available for the interpretation of results. The technique should be subject to the same quality control standards that other laboratory tests, and large validation processes will be necessary. We, laboratory specialists, should take advantage of our privileged, transversal position and robust technical knowledge to optimize the use of MS in patient care flow, which will contribute to improving clinical and prognostic evaluation.
